# Effect of Whey Proteins on Malnutrition and Extubating Time of Critically Ill COVID-19 Patients

**DOI:** 10.3390/nu14030437

**Published:** 2022-01-19

**Authors:** Marialaura Scarcella, Emidio Scarpellini, Alessandra Ascani, Rita Commissari, Claudia Scorcella, Michela Zanetti, Amilcare Parisi, Riccardo Monti, Natasa Milic, Abele Donati, Francesco Luzza, Edoardo De Robertis, Ludovico Abenavoli

**Affiliations:** 1 Anesthesia, Intensive Care and Nutritional Science, Azienda Ospedaliera “Santa Maria”, Via Tristano di Joannuccio, 05100 Terni, Italy; m.scarcella@aospterni.it; 2 Clinical Nutrition and Internal Medicine Unit, “Madonna del Soccorso” General Hospital, Via Luciano Manara 7, 63074 San Benedetto del Tronto, Italy; emidio.scarpellini@med.kuleuven.be; 3 Translational Research in Gastrointestinal Disorders (T.A.R.G.I.D.), Gasthuisberg University Hospital, KU Leuven, Herestraat 49, 3000 Lueven, Belgium; 4 Anesthesia and Intensive Care, Azienda Ospedaliera “Santa Maria”, Via Tristano di Joannuccio, 05100 Terni, Italy; a.ascani@aospterni.it (A.A.); r.commissari@aospterni.it (R.C.); 5 Clinic of Anesthesia and Intensive Care Unit, Azienda Ospedaliera Universitaria “Ospedali Riuniti”, Via Conca 71, 60126 Ancona, Italy; claudia.scorcella@ospedaliriuniti.marche.it; 6 Department of Medical and Surgical Sciences, Azienda Sanitaria Universitaria “Giuliano-Isontina”, Trieste University, 34148 Trieste, Italy; zanetti@units.it; 7 Emergency Department, Azienda Ospedaliera “Santa Maria”, Via Tristano di Joannuccio, 05100 Terni, Italy; a.parisi@aospterni.it; 8 Cardiovascular Intensive Care Unit, Surgical Unit and Recovery Room, Neonatal Intensive Care Unit, Obstetrics Operating Room, Azienda Ospedaliera “Santa Maria”, Via Tristano di Joannuccio, 05100 Terni, Italy; r.monti@aospterni.it; 9 Faculty of Medicine, University of Novi Sad, 21000 Novi Sad, Serbia; natasa.milic@mf.uns.ac.rs; 10 Department of Biomedical Sciences and Public Health, Università Politecnica delle Marche, Via Tronto 10/A, 60126 Ancona, Italy; abele.donati@staff.univpm.it; 11 Department of Health Sciences, University “Magna Graecia”, 88100 Catanzaro, Italy; luzza@unicz.it; 12 Anesthesia, Intensive Care and Pain Medicine, Università degli Studi di Perugia, Piazza Università, 06123 Perugia, Italy; edoardo.derobertis@unipg.it

**Keywords:** pre-albumin, C-reactive protein, extubating time, whey proteins, inflammation

## Abstract

The novel SARS-CoV-2 virus has led to a severe pandemic, starting from early 2020. Intensive care (ICU) management of the COVID-19 disease is difficult with high morbidity and mortality. Early nutritional support, especially with whey protein, seems to be crucial in this medical case. Thus, we aimed to assess the effects of an adequate nutritional protocol rich in whey protein on nutritional and inflammatory status, extubating time, and mortality of critically ill COVID-19 patients (CICP). Methods: A prospective single-center exploratory observational study was undertaken on 32 consecutive CICP admitted to the ICU of Santa Maria Hospital, Terni, Italy, and treated with whey protein-enriched formula. Patients’ demographics, nutritional status, indexes of inflammation, daily pre-albumin serum levels, duration of mechanical ventilation, and mortality were recorded. Results: Thirty-two patients were enrolled. Ninety-five percent of them showed a gradual reduction in C-reactive protein (CRP) values and increase in pre-albumin levels after the whey protein-enriched formula. Prealbumin levels were not correlated with a better nutritional status but with a shorter extubating time and better survival. Conclusions: An adequate administration of whey protein during COVID-19 patients’ ICU stays can provide fast achievement of protein targets, reducing the duration of mechanical ventilation, and improving inflammatory status and ICU survival. Further prospective and large-scale, controlled studies are needed to confirm these results.

## 1. Introduction

From January 2020, the novel corona virus (SARS-CoV-2) pneumonia (COVID-19) spread around the world, causing a tremendous global health emergency and, then, a long-lasting pandemic. This novel viral agent causes, in a significant percentage of the infected patients, a serious bilateral pneumonia that resembles severe acute respiratory syndrome (SARS) and Middle East respiratory syndrome (MERS). The severity of the clinical condition has been reported to be milder than SARS with a mortality rate ranging from 4.3% to 11% [1].

SARS-CoV-2 leads to a vast panel of different clinical manifestations. COVID-19 pneumonia is often associated with gastrointestinal disorders that compromise the patient’s ability to be adequately fed in the pre-Intensive Care Unit (ICU) stages [1].

The data from GiViTi (Italian Group for the Evaluation of Interventions in Intensive Care) show a direct correlation between inflammatory status, incidence of comorbidities, and mortality in these patients [2]. The main defensive mechanisms in human body against SARS-CoV-2 are the physical barriers, such as skin and mucosal membranes, stomach acid content and digestive enzymes, gut microbiome, and the acquired immunity, responsible for the production of several specific antibodies. Many of the reactions maintaining these mechanisms need vitamins A, D, B, iron, and zinc as coenzymes. This is one of the main reasons why setting an appropriate nutritional strategy as a part of the treatment is crucial for survival of these patients [2].

The inflammatory status of COVID-19 patients is associated with an increased mortality [3,4]. Moreover, the higher is the Nutritional Risk Score (NRS), the higher is the incidence of acquired healthcare associated infections and the mortality risk index [3,5]. The COVID-19 patient in the ICU is a frail patient with multiple comorbidities, affected by hypoxemia, inflammation, high body temperature, increased oxygen demand, and often prone to malnutrition. In the early stages of COVID-19, patients experience anorexia exacerbated by severe coughing, fever, dyspnea, anosmia, hypoxia, and fatigue, causing difficulties to maintain an appropriate nutritional oral intake. Moreover, before ICU admission, the patients are often treated with Continuous Positive Airway Pressure (CPAP) or Non-invasive Ventilation (NIV) that do not allow oral feeding in a large percentage.

Once admitted to the ICU, nutritional risk could be monitored through a timely and longitudinal prealbumin and C-reactive protein (CRP) levels trends [6,7]. In most cases, prealbumin levels are very low vs. those of CRP, showing a preexisting malnutrition and inflammatory status in these patients, increasing their nutritional risk before ICU admission [8,9,10,11,12]. Pre-albumin is not a selective and specific nutritional marker as it can be used as an indicator of both nutritional and inflammation risks. Indeed, its value is inversely related to the inflammation status regardless of underlying nutritional status [12]. In addition, normalization of pre-albumin levels can be used as an index of the resolution of inflammation, reduction in nutritional risk and transition to anabolism [12].

In the ICU, the COVID-19 patients are mechanically ventilated for several days, turned into a prone position, then passed to non-invasive techniques, causing a sarcopenic and myopathy status [13]. Thus, a specific nutritional protocol is fundamental for this kind of patients [13,14,15]. Specifically, high nitrogen, concentrated in volume to be easily adsorbed formula enriched with anti-inflammatory elements like omega-3 fatty acids and whey proteins is preferable to feed these patients [13,14,15].

This prospective observational exploratory single-center study aims to evaluate the nutritional and anti-inflammatory effects of an outlined nutritional protocol based on enteral nutrition (EN) with a whey protein-based formula in COVID-19 patients admitted to the ICU. In detail, we aim to verify improvement of the nutritional risk and pre-albumin trends after this formula administration. Moreover, we aim to study the possible correlation between the pre-albumin levels and the duration of mechanical ventilation and mortality.

## 2. Materials and Methods

### 2.1. Study Protocol

In this single-center perspective exploratory study, we consecutively enrolled 32 COVID-19 adult patients admitted at the ICU of “Santa Maria Hospital“, Terni, Italy, between 9 March and 30 April 2020. We respected regional Ethical Committee rules for patients’ enrollment (Ethical Committee CEAS Umbria, Italy, provisional CER N3650/20, April 2020). Inclusion criteria were: age > 18 years, confirmed diagnosis of SARS-CoV-2 infection, and need of mechanical ventilation for at least 24 h. Patients were treated according to the present guidelines for COVID-19 [16].

### 2.2. Nutritional Strategy Scheme

According to a standardized nutritional protocol for COVID-19 patients, each patient received EN applying the following nutritional scheme [17,18]:

- Starting of EN within 24 to 48 h from ICU admission, aiming to cover 50% of caloric needs;
- Ramping up to 80–100% of caloric needs after 48 h;
- Aiming a protein target ≥ 1.3 g/kg/day [17,18];
- Aiming a caloric target of 20–25 kcal/kg/day;
- Using high-protein, 100% whey proteins, casein free, low in carbohydrate (CHO) content, and with a fat/CHO ratio of 50/50 EN formula, rich in Medium-Chain Triglycerides (MCT) and omega-3 fatty acids, with a complete vitamins, minerals, and trace elements profile.

EN was also administered to the patients during prone positioning, maintaining at least 10% of bed anti-Trendelenburg tilt, and to the patients treated with NIV and in the conditions of controlled hypoxemia and permissive hypercapnia. EN was started at 20 mL/h infusion rate and increased by 10 to 20 mL/h every 24 h, based on the gastric tolerability. The feeding rate was reduced in case of >500 mL/24 h, adding pro-kinetics (i.v. metoclopramide 10 mg t.i.d.), and evaluating the correct positioning of the nasogastric tube and the needs of supplementary parenteral nutrition. In case of 1% propofol infusion higher than 20 mL/h, the calories and amount of lipids provided with this drug were taken into account in the daily energy balance. Concerning the obese patients, nutritional targets were calculated considering Body Mass Index (BMI) and Ideal Body Weight (IBW) as follows:

Calories:
  11–14 kcal/kg current body weight/day, for BMI of 30–50;
  22–25 kcal/kg IBW, for BMI > 50;
  65–70% of the measured calories.
Protein intake:
  2 g/kg IBW for BMI 30–50;
  2.5 g/kg IBW for BMI > 50.

The needs of trace elements and vitamins supplementation were evaluated in previously malnourished patients, or in case of partial EN with or without complementary parenteral nutrition (administered 250 mL of 5% glucose solution in 5 to 6 h during the night-time, once a day, for three days as a loading dose, and then twice a week in the maintenance dose, respectively) [17,18].

### 2.3. Indirect Calorimetric Measurements

The mREE data were obtained using the Q-NRG+^®^ Metabolic Monitor (Baxter, Rome, Italy). Patients admitted to ICU were temporarily excluded from indirect calorimetry (IC) assessment under the following conditions: fraction of inspired oxygen (FiO_2_) > 70%, hemodynamic instability, positive end-expiratory pressure (PEEP) > 16 mmHg on active veno-venous extracorporeal membrane oxygenation (VV ECMO), or per ICU attending clinical judgment. IC data were selected from 10 to 30 min intervals, which met steady-state conditions defined by a variance of volume of oxygen (VO**_2_**) and volume of carbon dioxide (VCO**_2_**) by <10% as per published validation data for the Q-NRG device [19]. The patients with multiple IC measurements were averaged over weekly intervals.

### 2.4. Data Collection 

All the clinical and laboratory data were prospectively collected from the patient’s medical file. General and demographic variables on the day of ICU admission were recorded and the SAPS II score was calculated in order to obtain the information on the severity of critical illness [20]. All the other variables were recorded daily for the entire ICU stay, starting from admission to discharge/death: pre-albumin serum, HDL and LDL cholesterol, inflammation and infection markers (CRP, IL-6, white blood cells count, and formula, procalcitonin, and erythrocyte sedimentation rate), renal and hepatic function indices, and blood gas analysis variables. The main evaluated outcomes were the ICU mortality and the duration of mechanical ventilation (extubation time). All the collected data were included in a database warranting the anonymity of the patients and using numbers as identification code for the subjects.

### 2.5. Statistical Analysis 

Statistical analyses were performed using SPSS Software 21 (IBM, New York, NY, USA). Quantitative variables distribution was tested with Kolmogorov–Smirnov normality test. All data are presented as mean ± standard deviation (SD) or median (interquartile range, IQR) according to the normal or not normal distribution. Parametric (Student’s T-Test) and non-parametric tests (Mann–Whitney U test) were applied to describe the differences between groups for the variables of interest as appropriate. Kaplan–Meier survival curves for the comparison of the Hazard Ratio between groups were built for the survival analysis. The alpha level of significance was set at 0.05 [21].

## 3. Results

From 9 March to 30 April 2020, 32 ICU SARS-CoV-2 infected patients meeting the inclusion criteria were consecutively enrolled. The study population had a mean age of 68 ± 12.5 years, with a mean SAPS II score of 57 ± 12 points at the admission to the ICU. Considering inflammatory/infection indices at day 1, median procalcitonin was 0.37 (0.19–1.29) ng/mL, median CRP was 19 (5.6–31) mg/L; white blood cells count 8070 (6263–11,000). ICU mortality was 31.3% (10 patients). Non-survivors were significantly older and severely ill as shown in Table 1. Median pre-albumin serum level at the ICU admission was 9 mg/dL [6,7,8,9,10,11,12,13,14].

Because of high formula tolerability, it was possible to administer all the patients with the following average intake: mean daily caloric intake: 20 to 30 kcal/kg/day; mean daily protein intake: 1.3 g protein/kg/day.

The first section of the analysis was focused on pre-albumin values during the ICU in the study population. Median serum pre-albumin level at the ICU admission was 9 (range 6–14) mg/dL. The 59.4% of the patients (19 out of 32) had a pre-albumin level below 10 mg/dL threshold at the ICU admission, as a possible sign of pre-existent state of malnutrition. This finding was only partially in agreement with indirect calorimetry assessment that showed a mild tendency toward hypermetabolism (21.3 ± 1.0 kcal/kg/d). There was no statistical difference between survivors and non-survivors (*p* = NS) (Table 1). This value included both obese and non-obese patients; the latter showed a significant difference in terms of hypermetabolism tendency (22.2 ± 1.2 vs. 27.0 ± 1.3 kcal/kg/d, obese vs. non-obese patients, *p* < 0.05).

**Figure 1:**
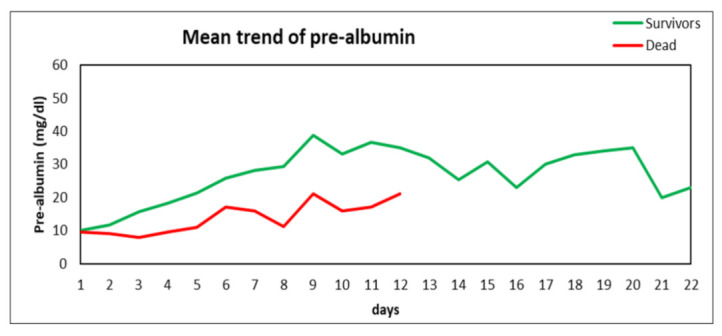
Mean serum pre-albumin levels trend of survivors and non-survivors during days of ICU stay (*p* < 0.05).

Twelve patients (63.2%) were able to reach and maintain the pre-albumin threshold during the ICU stay, while one patient reached that at a certain point of the ICU stay but did not maintain that. Six patients (31.6%) did never reach the threshold despite the application of the nutritional protocol. In all the 32 analyzed patients, there was a trend toward increase in pre-albumin levels during the ICU stay. This trend was not shown in three patients belonging to the group of non-survivors (survivors vs. non-survivors, *p* < 0.05) (Figure 1).

**Figure 2:**
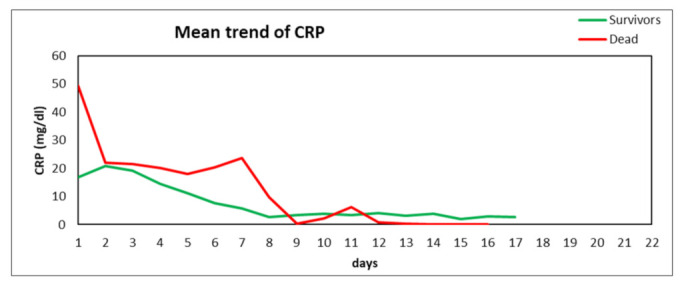
CRP trend during ICU stay of survivors and non-survivors (*p* = NS) during days of ICU stay. NS: not significant.

On the other hand, a decreasing trend in CRP was observed, both in survivors and non-survivors (*p* = NS) (Figure 2). Other inflammatory markers (e.g., IL-6 levels) followed CRP trend (data not shown).

**Figure 3:**
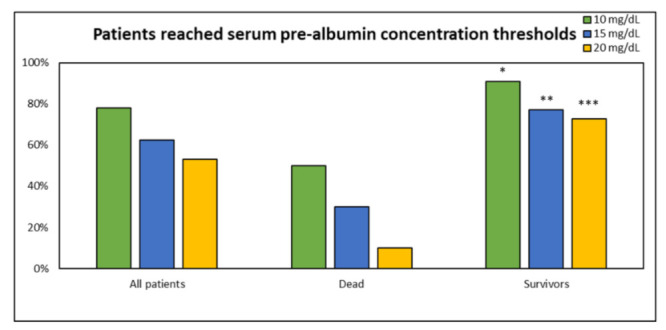
Percentage of patients reaching different serum pre-albumin concentration thresholds. All patients’ data are presented firstly, and then data on non-survivors and survivors. There was not statistical difference between percentages of patients reaching different PT (*p* = NS). A significantly lower percentage of non-survivors was able to reach the considered pre-albumin thresholds vs. survivors (*, **, ***, all *p* < 0.05). This was not observed in non-survivors’ group (*p* = NS).

In order to investigate the percentage of the patients reaching different thresholds of serum pre-albumin concentration during their ICU stay, the threshold was defined as reached: if at least 75% of subsequent measurements were not below the threshold itself, then it was assumed that the patients had maintained the considered threshold. In detail, 81.3%, 62.5%, and 53.1% of the study population reached the threshold of 10 mg/dL, 15 mg/dL, and 20 mg/dL, respectively (*p* = NS) (Figure 3). Interestingly, a significantly lower percentage of non-survivors was able to reach the considered pre-albumin thresholds vs. survivors (*p* < 0.05). This was not observed in non-survivor group (*p* = NS).

**Figure 4:**
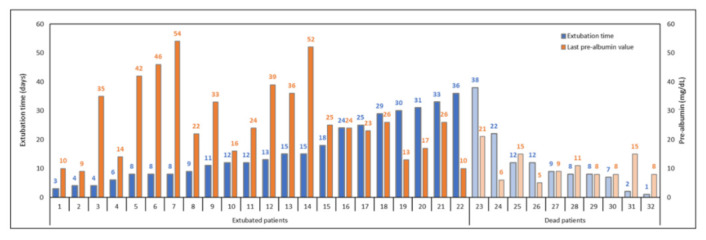
Extubation time and pre-albumin value of survivors and non-survivors during the days of ICU stay: Patients are represented in ascending order of extubation time (for survivors) and in descending order of survival time (for non-survivors). For each patient the last pre-albumin value available before the event (extubation or exitus) is represented. A decreasing trend is observed in pre-albumin level of extubated patients (high levels of pre-albumin—reduced extubation time), while for deceased patients the trend is more fluctuating.

The Figure 4 describes the association between pre-albumin levels and the duration of mechanical ventilation and ICU mortality, respectively: patients are represented in ascending order of extubation time (for survivors) and in descending order of survival time (for non-survivors). The last pre-albumin value available before the event (extubation or exitus) is represented for each patient. The patients with a shorter mechanical ventilation and successful extubation showed a tendency to have higher serum pre-albumin levels vs. non-survivors.

**Figure 5:**
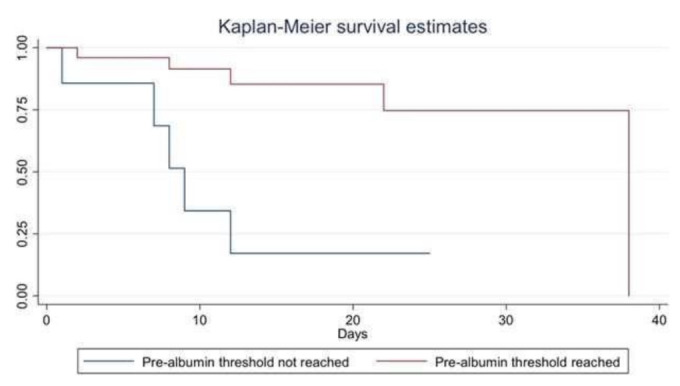
Comparison of survival of patients who reached and maintained the pre-albumin threshold (in green) and patients failing to reach and maintain it (in red). The patients who were able to reach and maintain the serum pre-albumin level threshold > 10 mg/dL showed a greater ICU survival (Log-rank test, *p* < 0.00001).

Moreover, if a successful extubation was reached within 14 days, the 10 mg/dL pre-albumin threshold seemed to be a positive predictive factor (42.3% vs. 16.7%, OR = 3.67 (0.37–35.98). Finally, looking at the survival analysis following Kaplan–Meier, the patients who were able to reach and maintain the serum pre-albumin level threshold > 10 mg/dL showed a greater ICU survival vs. other patients (Log-rank test, *p* < 0.00001) (Figure 5).

**Figure 6:**
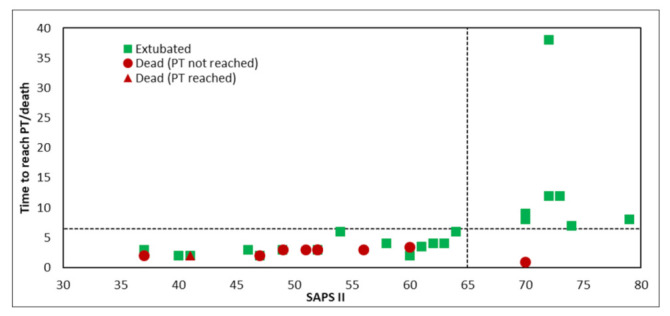
Association between SAPS II score and time to reach protein target (PT).

All surviving patients reached a target of protein intake of 1.3 g/kg/day. The median achievement time was 3 ± 0.3 days (range 2–6). Only one patient in the “non-survivors” group could reach the protein intake target; the target time was of 3 ± 0.4 days. The most severely ill patients (identified by a higher SAPS II) appeared to require a longer time to reach the protein target (PT) (Figure 6). Interestingly, the patients reaching the PT, each 5-point increase in the SAPS II score was correlated with an average delay in the target time of 11.1 h (95% IC 6.8–15.5; *p* < 0.0001).

**Figure 7:**
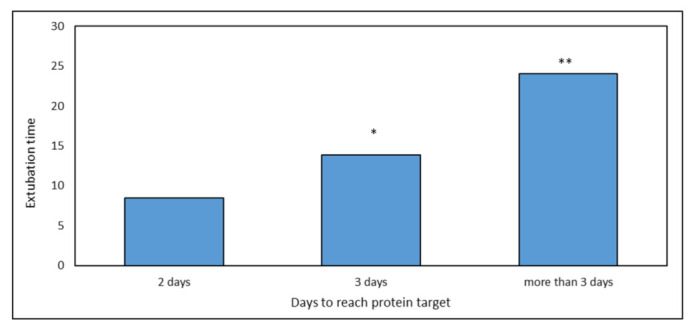
Extubation time related to different PT thresholds. There was a significant correlation between time to reach PT and extubation time (*, **, both *p* < 0.05).

Finally, a positive correlation was observed between time to reach the PT and extubation time (*p* < 0.05) (Figure 7).

## 4. Discussion

The present prospective observational study has primarily been aimed to investigate the impact of whey protein-based EN formula on nutritional, inflammatory markers, extubation time, and survival rate of SARS-CoV-2 critically ill patients. Pre-albumin values significantly increased, and this was followed by CRP reduction and a better survival rate, rather than nutritional status. The application of a feeding protocol based on whey protein-based formula seems to offer a survival advantage to these patients. In detail, the majority of the patients were able to reach the protein intake target and the time to reach this target showed an association with the extubation time. Moreover, this EN regimen allowed to reach normal serum pre-albumin levels in a vast majority of the study population and this was associated with lower ICU mortality and a reduction in the duration of mechanical ventilation.

However, IC data did not agree with pre-albumin levels during the ICU stay. Indeed, pre-albumin is a controversial marker of malnutrition and nutritional risks both in COVID-19 and non-COVID-19 patients [22]. Malnutrition is characteristic of the ICU patients [23]. Acute illness makes a patient unable to move, to have an oral food intake. Muscle wasting and malnutrition are typical of the ICU patient of any cause. Advanced age, comorbidities, together with preexisting malnutrition, common to critically ill patients can contribute to malnutrition [24]. Malnutrition is significantly associated with poor outcomes including increased mortality of ICU patient. Thus, nutritional support is crucial for critically ill patients: early, progressive EN formulas enriched in proteins seem to guarantee a better survival in COVID-19 and non-COVID-19 critically ill patients [25]. Therefore, we can consider these patients, especially in the case of the new SARS-CoV-2 infected subjects, as fighting “bodybuilders“ needing an adequate and high protein intake due to the hypercatabolic state of COVID-19 [26]. However, monitoring the need for nutritional support and relative response to it, is challenging. Society nutrition guidelines and recent metanalyses [27,28] have highlighted how the changes in body weight and its composition are not reliable biomarkers of nutritional state for these patients because of their extensive hydration and catabolism. Moreover, metabolic status indexes such as nitrogen balance and protein turnover are difficult to assess in clinical practice because of the risks and complexity of measurements [28]. To date, indirect calorimetry is the gold standard to define and monitor nutritional status and metabolism of a critical patient [29]. However, few ICUs have IC available, and its usage is energy-consuming and sometimes harmful, especially in COVID-19 patients.

Pre-albumin is a tetrameric protein secreted mainly by the liver and choroidal plexus and accounts for 15% albumin serum level (namely, thyroid hormone transport). Due to pre-albumin short half-life (2 days) vs. albumin (14–20 days), small volume of distribution and uncomplicated measurement, it could be a good candidate for monitoring rapid changes in metabolic status [30,31]. Several studies have described pre-albumin being a negative acute phase reactant as inflammatory cytokines reduce its hepatic synthesis. Therefore, serum concentrations may decrease despite sufficient nutrition supplementation or absence of malnutrition, especially in critically ill patients [32]. In fact, visceral proteins, such as pre-albumin, also reflect the dynamic and catabolic response to surgery, stress, injury, infection, and organ dysfunction [33]. Indeed, serum pre-albumin levels show a promising and practical utility as a prognostic index in critically ill patients [34]. Furthermore, this trend has been used as a tool to monitor the nutritional status of critical and non-critical patients.

In this study population, a relevant number of the patients was admitted to the ICU with low serum pre-albumin levels. This could be interpreted as a sign of diffuse malnutrition status in these patients even before the ICU admission. However, we must recognize that COVID-19 patients arrive at the intensivist attention after days of illness, treated both at home and in general wards. More in detail, COVID-19 is often associated with fever, lack of appetite, and various gastrointestinal disturbances such as nausea, vomiting, and diarrhea. These conditions can bias and influence the values of pre-albumin at the moment of ICU admission. Other biasing factors are the large use of CPAP masks and NIV that can substantially contribute to malnutrition and muscle waste before the ICU admission. Furthermore, severe protein degradation may lead to unintended and extensive muscle wasting (up to 15–25% of the muscle mass), recently quantified during the first ten days after ICU admission [19]. Thus, the pre-albumin values detected in this study describe both the severe inflammatory state and the impaired nutritional status of patients. However, we must recognize that there was no significant correlation between pre-albumin values at admission and nutritional status measured by IC.

In this study, we observed that a whey protein-enriched nutritional scheme can reverse the vicious circle of protein depletion related to both ICU admission and COVID-19 clinical course. In a prospective database of 843 ICU patients undergoing mechanical ventilation [18], a significant progressive reduction in mortality rate (from 36% to 19%) was associated with an additional protein daily intake starting from 0.8 to 1 g protein/kg bw/day up to ≥1.2 g protein/kg bw/day (*p* = 0.033). Scientific society guidelines and recommendations are in favor of this practice. More in detail, a sufficient (high-dose) protein load should be provided to critically ill patients with a protein requirement expected to be in the range of 1.2–2.0 g/kg actual body weight per day. These aspects should be taken into great account when approaching COVID-19 patients and their nutritional status and risk of rapid malnutrition development. Moreover, high-protein formulas and protein derivatives can have direct anti-viral and anti-inflammatory effects in COVID-19 [35]. The vast majority of the patients in this study population were able to reach a normal threshold for serum pre-albumin levels. This suggests that the used formula has a high tolerability and efficiency in achieving nutritional targets minimizing the malnutrition risk and reducing the inflammatory response typical for COVID-19 and non-COVID-19 ICU patients. The conceivable effect on nutritional status of patients is supported by achieving a good balance in energy/proteins ratio and by improving IC data of a subset of patients. In fact, we were able to reach the protein target of 1.3 g/kg bw/day in an average time of 3.2 days (76.8 h) in the majority of patients (the less severe ones, SAPS II < 65). On the other hand, in the most severe ICU patients, (SAPS II > 65), the timing to reach the protein intake target was directly correlated with SAPS II values (11.1 h more every 5 points of SAPS II values).

In literature, there are different findings on the efficacy of a high-protein formula in reaching such results on mortality and morbidity. Alberda et al. carried out a multicenter study on more than 2770 ICU mechanically ventilated patients [36]. Interestingly, the average daily coverage of nutritional prescription was only 59.2% for energy and 56% for protein, respectively. The reasons for this insufficient nutritional need coverage can range from feeding withdrawal required by clinical exams and blood tests to reduced nutritional intake due to poor gastrointestinal tolerance or metabolic problems. Gungabisson et al., described GI intolerance occurring during EN in critically ill patients: they registered an incidence of intolerance of 30.5% occurred after a median of 3 days upon EN initiation [37]. The patients remained intolerant for a mean duration of 1.9 ± 1.3 days; GI intolerance was associated with poorer nutrition and clinical outcomes. Intolerance was associated with worse nutrition adequacy (56% vs. 64%, intolerant vs. tolerant patients, respectively, *p* < 0.0001), increased ICU stay (14.4 vs. 11.3 days, intolerant vs. tolerant patients, respectively, *p* < 0.0001), and increased mortality (30.8% vs. 26.2%, intolerant vs. tolerant patients, respectively, *p* = 0.04) [19]. We have chosen whey protein for our standardized EN formula for several reasons. From a metabolic point of view, whey proteins are classified among the ones with the highest biological value due to their high concentration of essential amino acids. As “fast proteins”, they are digested and metabolized much faster than caseinates and, therefore, provide better and more efficacious amino acid uptake for muscle anabolism. Their high cysteine content makes whey proteins one of the best sources of these amino acids for the proper functioning of glutathione peroxidase, and therefore increasing the body’s ability to exert clearance of free radicals and reduce their toxicity [38].

In this study, the pre-albumin trend was significantly correlated to an increased nutrition and mortality risk [12]. The visceral proteins, namely, albumin, transferrin, and pre-albumin, must be correctly recognized as inflammatory markers associated with “nutrition risk” in nutritional assessment. In fact, the CRP trend values decreases vs. the increasing trend of pre-albumin in the same patient population, both in survivors and non-survivors, as shown in Figure 5. These results can be of particular relevance in COVID-19 patients characterized by hyper-inflammatory and catabolic states [39]. In the present study, other steps of the ICU stay were significantly correlated with serum pre-albumin levels: extubation time of patients, which reached higher pre-albumin values, was found to be lower vs. those not-reaching such values. In addition, reaching a pre-albumin > 10 mg/dL appeared to be a positive cut-off for successful discontinuation of mechanical ventilation within 14 days of the ICU stay. This finding has to be confirmed by larger studies. Finally, last but not least, the achievement of higher pre-albumin values seems to be associated with a better survival outcome. All patients with early ICU mortality showed pre-albumin values < 10 mg/dL. This finding is in agreement with some studies from COVID-19 and non-COVID-19 literature and needs larger controlled trials to be confirmed [34].

This study has several limitations to be acknowledged. The first, and main limitation, the single-center exploratory design does not allow the authors to draw definitive conclusions regarding the real impact of this EN whey protein-enriched protocol in COVID-19 patients. An interventional control group is missing, perhaps one using a different EN formula. This has been conditioned by the need to provide the best EN protocol to COVID-19 patients in this pandemic. Second, the small sample size and lack of a control group (e.g., non-COVID-19 patients) do not allow the authors to establish a direct causality connection between the investigated variables and outcome. Third, pre-albumin is not a reliable nutritional status biomarker, and findings from the present study confirm this evidence. On the other hand, pre-albumin has been confirmed as a “hybrid” biomarker for mortality and morbidity of ICU patients. Findings from this study need to be confirmed by larger multicentric controlled trials.

## 5. Conclusions

In conclusion, the application of a nutritional protocol with a highly tolerated enteral formula based on whey protein, suggests a positive effect on patients’ outcome and a positive effect on the duration of mechanical ventilation in SARS-CoV-2-infected critically ill patients. Pre-albumin seems to be useful in survival rate prediction in ICU patients.

The anti-inflammatory properties of whey proteins could have an important adjunctive role in the treatment of high inflammation-generating infections such as the new SARS-CoV-2 virus. These preliminary results will ensure further larger prospective controlled investigations in this field.

## Figures and Tables

**Table 1:** Characteristics of the study population and outcome.

	Patients (n)	Age (years)	SAPS II	IC (mRee, kcal/kg/d)	Mortality
All patients	32	68.0 ± 12.5	57.3 ± 12.0	21.3 ± 1.0	31.3%
Non survivors	10	77.7 ± 8.7 *	69.9 ± 7.9 **	25.4 ± 1.2	NA
Survivors	22	63.5 ± 11.5	51.5 ± 8.8	22.1 ± 1.1	NA
*p*-value		<0.05	<0.05	NS	NA

Table legend: *, **: both *p* < 0.05; NA: non-applicable; NS: non-significant; IC: indirect calorimetry. Non-survivors were significantly older than survivors and had a worse SAPS II score (severity of illness) (* and **, both *p* < 0.05). Indirect calorimetry data showed no statistical difference in metabolism of surviors vs. non-survivors (*p* = NS).

## Data Availability

Data supporting these results can be found in the patients’ files database of “Santa Maria Hospital, Terni, Italy”.
